# AI applied to Saudi Arabia higher education: systematic literature review—SLR with PRISMA and VOSviewer

**DOI:** 10.3389/fdata.2026.1846964

**Published:** 2026-07-22

**Authors:** Jehad Alqurni

**Affiliations:** Department of Educational Technologies, College of Education, Imam Abdulrahman Bin Faisal University, Dammam, Saudi Arabia

**Keywords:** artificial intelligence, bibliometric analysis, higher education, PRISMA analysis, VOSviewer

## Abstract

This study explored the role of artificial intelligence in higher education in Saudi Arabia. It aims to identify trends, key contributors, influential papers, collaborations, and important research areas from January 2022 to February 2026 to guide future research. The researchers used bibliometric and content analyses. This combined quantitative descriptive methods and network analysis with qualitative content analysis of the most-cited articles. They extracted data from Scopus and the Web of Science, resulting in 66 documents after removing duplicates, editorials, and notes. The analytical techniques included the Preferred Reporting Items for Systematic Reviews and Meta-Analyses (PRISMA), co-word analysis, citation analysis, co-authorship analysis, and bibliographic coupling. VOSviewer supported the visualization. The key findings show that King Abdulaziz University, Qassim University, and King Saud University are the top contributors. A total of 52 papers have been published in journals indexed by WOS and Scopus, while 14 papers are indexed only by Scopus. Additionally, five major groups revealed significant correlations among various word pairs: High-education, Artificial intelligence, AI Chatbot, learning systems, and ChatGPT. The leading journals include Sustainability, Acta Psychological, and Systems, with notable authors such as Al-Harbi. Content analysis highlights the potential of AI to improve learning, boost administrative efficiency, and drive innovation in Saudi Arabia. This study offers practical recommendations for students, universities, and policy makers. This study has several limitations that should be addressed in future studies.

## 1 Introduction

The integration of artificial intelligence (AI) in Saudi Arabia's higher education sector supports the nation's 2030 Vision for Comprehensive Development. It plays a key role in changing the educational strategies and outcomes. Research shows that AI in Saudi higher education is still in its early stages but is considered an important reality for meeting future learning challenges in the country. The use of AI tackles significant educational issues by changing teaching methods, speeding up progress toward national development goals, and highlighting the need for students to gain the technical skills required to work with and develop AI technologies (Alotaibi and Alshehri, 2023). Stakeholders in Saudi higher education are aware of the potential of AI to improve teaching and learning experiences. They expect that it will also streamline administrative duties while promoting innovation. Despite(this), there is a sense of hope in the air about careers for any variety of learners and how AI can provide them with support infinitely long. Nevertheless, great attention should be paid to issues of privacy, security, and bias in AI's implementation, which could be damaging. Even knowing about AI integration must take into account technical, ethical, social, and educational factors for the responsible application of this technology to take root in academic circles (Al-Zahrani and Alasmari, 2024). For example, in academic fields such as architectural education, surveys show that while students are eager to adopt and familiarize themselves with AI tools, the faculty are hesitant. Some of this resistance stems from a worry that the AI (associated) might affect creativity.

The two groups agreed that it is essential to include AI in future curricula. In order for AI To enhance and encourage creativity in architectural learning and teaching, it is suggested that students and educators participate in training and workshops related to AI skills (Alshahrani and Mostafa, 2025). In addition, data-driven approaches, such as machine learning and big data, signify the future of AI implementation, which can facilitate personalized learning and better decisions by institutions. Random Forest predictive models have been shown to be highly accurate and beneficial for the educational process in their uses. Nonetheless, realizing the benefits easily and sustainably will require overcoming challenges, such as high costs, privacy concerns, and a shortage of qualified personnel (Khan et al., 2025). The acceptance of AI among students is influenced by usability factors. This refers to factors in digital learning platforms, such as LMS. There are factors that influence the perceived usefulness and PEU of the system. These include quality content, system navigability and interactivity, and instructional support. Given that LMS is still growing in Saudi higher education, improving these components can enhance students' engagement with AI-powered educational tools (Binyamin et al., 2019).

The Saudi architectural education case study provides a statement of students' enthusiasm vs. faculty's reluctance. Students demonstrated a high level of interest and understanding of the use of AI tools, whereas the faculty members were skeptical, fearing that excessive use of AI might undermine creativity and creativity phases, commonly known as “design shallowing,” where they choose to use AI-generated options instead of going through the design thinking process (Alshahrani and Mostafa, 2025). The rigidity of AI frameworks to limit creative thinking is also cited in a wider-scale study of AI in college education in relation to the applications of AI in teaching (Lin and Chen, 2024). One of the strengths of RF is its ability to manage numerous variables, minimize overfitting, and pinpoint the most significant predictors of the problem, such as the number of units taken, grade in subjects, use of library, and frequency, which can then be used to inform interventions and allocation of resources (Remegio, 2024; Saputri et al., 2025). In all Technology Acceptance Model (TAM) studies, Perceived Ease of Use (PEU/PEOU) reflects the ease with which users perceive a system to be learned and used by them. Generally, it is beneficial for enhancing attitudes, satisfaction, and intention to use AI-based e-learning (Saqr et al., 2023; Li et al., 2024). AI supporting personal learning environments (PLE) has a significant influence on perceived ease of use and perceived usefulness, which consequently impacts attitude, satisfaction, and intention to use e-learning, which varies according to gender and learner type (Kashive et al., 2020). In a related study on AI-driven platforms, social learning networks and personal learning portfolios were shown to increase perceived usefulness and ease of use, and self-efficacy and innovativeness acted as moderators of intentions (Saqr et al., 2023).

Systematic reviews have identified the transformative effect of ChatGPT in Saudi higher education through supporting teaching, increasing student engagement, and improving research and writing proficiency, language learning, and educational inclusivity. The instrument fits the objectives of Saudi Arabia's Vision 2030 which focuses on academic excellence and workforce readiness, and puts forward the idea of ChatGPT as a device capable of reducing the language barrier and providing individualized assistance (Faisal, 2024). Therefore, a conceptual framework based on Task-Technology Fit (TTF) further highlighted that a perfect fit between the learning tasks of the students and the abilities of ChatGPT is essential in increasing the behavioral intentions of the students to use the technology. This study emphasizes the need to ensure that AI tools are strategically integrated into educational activities to enhance their usage and learning outcomes in Saudi higher education institutions (Al-Mamary et al., 2024).

Worldwide, the field of education is undergoing a transformation driven by Artificial Intelligence (AI), presenting a new array of opportunities to enhance pedagogical practices, personalize learning, streamline administrative processes, and inform institutional decision-making. Saudi Vision 2030 and the National Strategy for Data and AI (NSDAI) are among the many initiatives in the Kingdom of Saudi Arabia that contribute to this global transformation. In this context, national policies highlight the need to integrate AI into the HE sector to boost the HE ecosystem and foster universities to become modern, technologically advanced, and capable of producing a future-ready workforce (Alotaibi and Alshehri, 2023; Alsahafi and Baashar, 2025). Despite this strong strategic support, empirical studies suggest that the use of AI in HE in Saudi Arabia is still in its early stages. Existing Nationwide surveys show a huge gap between students' interest and institutions' preparedness. Saudi university students often use generative AI tools and are moderately ready technically, but their interactions with these tools are not formal or part of the curriculum; rather, they are done on their own initiative (Alabbad et al., 2025; Al Shahrani et al., 2024). Moreover, the level of readiness and exposure to the use of AI technologies is very heterogeneous and is strongly affected by demographic variables such as gender, geographic region, and academic field (Alotaibi and Alshehri, 2023; Almusharraf et al., 2025). However, the integration of AI in Saudi universities currently faces three sets of challenges: institutional, attitudinal, and pedagogical. However, although students demonstrated a positive attitude toward the impact of AI on their future careers, they also voiced concerns about the impact of job changes and the quality of their education (Aboalshamat, 2022). Recent research has repeatedly reported a high level of faculty member resistance, pedagogical unpreparedness, and low levels of faculty member AI literacy, as well as digital infrastructure gaps between large urban and regional universities (Alsahafi and Baashar, 2025; Almusharraf et al., 2025). Although Fadlelmula and Qadhi (2024) offered a general conceptual understanding of the challenges of implementing AI in the education sector in the GCC region, the literature to date fails to explain the specific and empirical realities of the Saudi Arabian context in which higher education is enacted. At present, several gaps in the literature need to be addressed.

1. Past reviews provide general insights into the GCC region but do not provide localized and empirical data on the impact of particular policies and cultural contexts in Saudi universities on the adoption of AI.
2. Lack of Standardized AI Curriculum: Non-technical majors do not have sufficient accredited and standardized AI curricula in Saudi universities.
3. The faculty development void: Although faculty unreadiness has been identified as a barrier, no faculty development programs specifically designed to enhance pedagogical AI literacy have been empirically tested.
4. Limited Methodology: Many current studies are cross-sectional surveys, while a long-term perspective on the practical implications of structured AI use on student learning outcomes and critical thinking remains absent.

Based on two points–the strategic importance of Vision 2030 and the current disjointed situation of AI implementation–the problem becomes evident: Saudi universities are at risk of the gap between top-down national-level AI policies and bottom-up university-level implementation.

In summary, Saudi educational research evidence suggests that ChatGPT has significant potential to improve higher education by promoting learner autonomy, language and academic skills development, and educational innovation. The present research is descriptive, network analysis, and content analysis, providing directions for further research. The following are the main questions discussed in this study: According to Alotaibi and Alshehri (2023), a bibliometric analysis based on their work can be conducted on the number, growth over time, and distribution across institutions and themes (Alotaibi and Alshehri, 2023); AI-based learning outcomes and institutional contributions (King Abdulaziz University, others) (Alotaibi and Alshehri, 2023); digital transformation projects in the Ministry of Education and critical success factors (Alojail et al., 2023; Alotaibi, 2022). Therefore, a better RQ1Q is as follows:

- RQ1Q: What are the publication trends and domain-specific applications of AI in Saudi Arabia's higher education sector?

The reporting of top authors, authors' institutions, top journals, and the most cited works has already been modeled in global bibliometric studies (Kavitha et al., 2024; Kavitha and Joshith, 2024; Maphosa and Maphosa, 2023). Identify and list the top Saudi (or Saudi-affiliated) authors/institutions/journals for HE AI and, where applicable, specify the main domain of their research [e.g., medical education (Alsahafi and Baashar, 2025)], pharmacy (Syed and Al-Rawi, 2023), architecture (Alshahrani and Mostafa, 2025), generic HE/GAI (Al-Zahrani and Alasmari, 2024; Alammari, 2024). Therefore, a minor adjustment of the wording for RQ2Q-RQ3Q was made.

- RQ2Q: What are the best contributing authors, institutions, journals, and countries of artificial intelligence in higher education in Saudi Arabia and by domain of higher education?
- RQ3Q: Which are the most cited articles on artificial intelligence, and how are they distributed across HE domains in Saudi Arabia?

Using VOSviewer, Alotaibi and Alshehri (2023) highlighted four themes in Saudi Arabia's AI-in-learning: new technology, big data, course/teacher/ability keywords, and regional work for the GCC, focusing on themes of adoption barriers and traditional teaching (Fadlelmula and Qadhi, 2024). Al Zahrani and Alasmari outline the ethical, social, and educational considerations regarding AI use in Saudi HE (Al-Zahrani and Alasmari, 2024). Thus, Saudi MoE digital transformation framework and success factors (Alojail et al., 2023), university-level digital and AI transformation projects (e-learning, sustainability, and Vision 2030 links) (Khan et al., 2025; Singh and Alshammari, 2023). Therefore, we rephrased the question for RQ4Q as follows:

- RQ4Q: What key subjects and themes structure AI-in-higher-education research in Saudi Arabia, and how do they relate to national and institutional initiatives (e.g., Vision 2030 and MoE digital transformation)?

Thus, to attain the expected outcomes of this study, two research objectives were established.

- The initial goal of the study was to fulfill the purpose of descriptive analysis, which is to present the necessary descriptive data and statistics in publications (background and theoretical framework of systematic literature review) according to the Preferred Reporting Items for Systematic Reviews and Meta-Analyses (PRISMA).
- The seconds aim of this study is to analyze and describe the expected interconnections between the various components of AI in Saudi Arabia's higher education research using VOSviewer.

## 2 Theoretical background

This research is about teaching assistants based on AI in higher education and poses the question of how features that resemble human beings and how they are perceived by students influence their readiness to use these technologies, with the theoretical framework being the Technology Acceptance Model (TAM) (Muneer et al., 2026). According to the authors, despite numerous values and principles of what should be considered trustworthy AI, there is a relative lack of consensus on what real issues should be of greater concern in enhancing software processes (SPI) in the context of creating AI systems (Akbar et al., 2024). Previous research on educational and Arabic chatbots has demonstrated possible advantages in motivation and support but also deficiencies in rigorous empirical testing and Arabic-language chatbots, which is why adaptive and static rule-based chatbots should be compared in a controlled study (Al Faia and Alomar, 2025). Current assessments of educational chatbots (and chatbots in general) are generally based on questionnaires (for example, SUS and UEQ) and *ad hoc* measures; thus, cross-study comparison is challenging, and there is a lack of joint assessment of engagement and effectiveness (Alabbas and Alomar, 2025). This is driven by the increased application of AI in education and research, as well as issues of accuracy, ethics, and responsible implementation of AI in academia and clinical practice (Alahmadi and Fadil, 2025; Syed et al., 2024).

AI chatbots are becoming a popular means of learning English, providing students with an opportunity to engage in conversational practice, which could be lacking in traditional classrooms (Alshayban, 2026). This study underlines issues in the context of mobile learning, specifically the problems of students with post-class reviews because of the lack of immediate support, which influences the engagement process and academic performance. It presents AI-powered ChatGPT as a possible way to improve mobile learning by offering on-demand and personalized academic support (Dahri et al., 2025a,b). The proposed study aims to identify interactions among gamification, AI, student engagement, and achievement in Malaysian educational settings through quantitative research (Dahri et al., 2025c). The research considers This study investigates the influence of several aspects on the intention to use ChatGPT for Metacognitive Self-Regulated Learning (MSRL) in a school environment (Dahri et al., 2024a). GenAI contributes to inclusivity, operational efficiency, innovative sustainability procedures, and risk recognition, including algorithmic bias, the digital divide, and high energy consumption (Dwivedi and Al-Banna, 2025). Research has examined the knowledge, use, and perception of AI chatbots among students in countries, including their potential to assist in learning activities, including studying and writing assignments, as well as raising concerns regarding accuracy and ethical concerns (El-Sobkey et al., 2025; Güler et al., 2026). It observes a general agreement on the central values of ethics, including fairness, transparency, accountability, explainability, and sustainability, but indicates a wide disparity in the implementation of these values by different regions and institutions (Ismail and Ahmad, 2025). Previous studies have largely concentrated on user preference and satisfaction regarding chatbot personality but not on behavioral outcomes (such as trust, engagement, and perceived authenticity) (Kuhail et al., 2022). However, the associated studies demonstrate that the overall perception of teachers toward AI is that it can assist personalized learning, classroom control, and inclusive education but raises concerns about ethical, digital literacy, and irreplaceable human qualities of teachers, such as emotional and social skills (Sharmin et al., 2026). This highlights the necessity of researching the mental, emotional, and situational conditions affecting the desire to delegate roles such as companionship, mental health advisor, doctor, and teacher to AI (Yankouskaya et al., 2026).

Unified Theory of Acceptance and Use of Technology (UTAUT) model to test the major predictors, including performance expectancy, effort expectancy, social influence, and enabling conditions that influence the adoption of generative AI tools by STEM students (BinJwair, 2025). BinJwair and Alamer (2025) placed their research in the context of the Unified Theory of Acceptance and Use of Technology (UTAUT) to comprehend the process of generative AI (GenAI) adoption by university students in Saudi Arabia. This study uses the SOR theory to model the effect of external stimuli (use of ChatGPT) on internal cognitive and knowledge processes (organism) that subsequently affect the response (e.g., academic success and career readiness). A model of technology acceptance is based on TAM constructs, which are applied to comprehend the factors of technology acceptance that could affect the use of ChatGPT among students (Dahri et al., 2025b). The UTAUT model is intended to determine the impact of performance expectancy, effort expectancy, information accuracy, pedagogical fit, and student interaction on AI adoption (Dahri et al., 2024b). The theoretical Framework relies on the hypothesis that ease of use, time and psychological risks, and TTF have a significant impact on the willingness of students to adopt AI based on technology acceptance theories to contextualize the relationships (Al-Mubireek, 2025). Activity Theory to describe how AI chatbots can be integrated into the educational system and how the combination of tools, rules, and division of labor can be more effective in improving the language learning process. It also implements social presence theory to examine how chatbots facilitate affective and coherent communication during language learning situations (Almusharraf, 2026). The theoretical frameworks for AI in Saudi Arabian education, task-technology fit (T-TF) theory, and the artificial intelligence device use acceptance (AIDUA) model were used to investigate the potential of MLLMs to increase performance benefits. Such frameworks can be used to determine the fitness of the technology for educational work and student acceptance of AI devices, which influence the readiness to use MLLMs (Al-Dokhny et al., 2024).

## 3 Research methodology

### 3.1 Overview of the systematic review protocol

This study adheres to the Preferred Reporting Items for Systematic Reviews and Meta-Analyses (PRISMA 2020) statement (Page et al., 2021) and PRISMA-S search extension guidelines (Rethlefsen et al., 2021) to ensure methodological rigor, transparency, and the ability to reproduce results. A structured literature review (SLR) architecture was developed to broadly address the applications, drivers, and challenges of AI in higher education in the Kingdom of Saudi Arabia (KSA). The use of this standardized framework helps reduce selection bias and offers an auditable path forward for future studies to be recreated.

### 3.2 Information sources and search strategy

To perform a comprehensive literature search, two bibliographic databases were chosen: Scopus (Elsevier) and Web of Science (WoS, Clarivate Core Collection), and this search was conducted on February 25, 2026. These platforms were chosen because they provide access to a wide range of indexed high-impact, peer-reviewed journals and conference proceedings in educational technology and computer science. Boolean operators and field tags were used to build specific search strings for the database by narrowing down the scope to artificial intelligence, higher education institutions, and the Saudi Arabian context to ensure geographic and thematic specificity while maximizing retrieval sensitivity.

To capture the most contemporary advancements and ensure relevance within this rapidly evolving field, the literature search was specifically bounded from January 2022 to February 2026. The exact search syntax, operational interfaces, and applied database filters are systematically detailed in Table 1 to ensure full transparency and reproducibility.

**Table 1:** Database search architecture and Boolean syntax strings.

Database interface/ platform	Exact search string syntax	Applied filters and metadata limits
Scopus (Elsevier)	TITLE-ABS-KEY (“Artificial Intelligence” OR “AI”) AND TITLE-ABS-KEY (“Higher Education” OR “University” OR “Universities” OR “Academic Sector”) AND TITLE-ABS-KEY (“Saudi Arabia” OR “KSA” OR “Saudi”)	Document Type: Article, Source Type: Journal, Language: English, Indexes: Scopus, Timeline: January 2022 to February 2026
Web of Science (Clarivate)	TS=(“Artificial Intelligence” OR “AI”) AND TS=(“Higher Education” OR “University” OR “Universities” OR “Academic Sector”) AND TS=(“Saudi Arabia” OR “KSA” OR “Saudi”)	Document Type: Article, Source Type: Journal, Language: English Indexes: SCI-EXPANDED, SSCI, AandHCI, ESCI Timeline: January 2022 to February 2026

### 3.3 Eligibility criteria

To set the boundaries of the concepts and empirical data, strict inclusion and exclusion criteria were defined before the screening process. The retrieved literature was judged based on relevance using these criteria.

**The inclusion criteria** were as follows: (1) the studies had to empirically or conceptually study the use of AI technologies in academic environments; (2) the studies had to be explicitly mentioned in public or private higher education institutions within the Kingdom of Saudi Arabia; (3) the studies had to be related to university students and faculty members; and (4) the studies had to be published as a peer-reviewed journal article in English.

**Exclusion Criteria:** The remaining 66 documents underwent a comprehensive full-text eligibility assessment based on strict, predefined exclusion criteria. Under these criteria, studies were subject to exclusion if they were related to general information and communications technology (ICT), data analytics, or e-learning platforms with no core AI, or dealt with primary or intermediate school (K-12 systems), or were aimed at corporate or industrial training outside of academia, or were secondary or non-empirical gray literature (editorial notes, trade magazines, book reviews, white papers, or extended abstracts). Upon rigorous evaluation, all 66 articles successfully fulfilled the eligibility requirements, resulting in zero (0) exclusions at this stage.

### 3.4 The screening, deduplication, and selection process

The selection process was carried out using a multistage workflow with a careful methodology and clear traceability of the records.

A total of 118 raw records were found with the initial search query on both databases, which included 32 records from Scopus and 86 records from the Web of Science. The bibliographic metadata files (in. ris formats) were downloaded and combined into one VOSviewer dataset for central processing. Duplication was at 41%, with the automated deduplication algorithm removing the overlap and leaving 77 unique records for title and abstract screening in the initial processing phase. The 11 excluded title/abstract records were carefully evaluated by the author against the predefined inclusion criteria and were subsequently excluded.

The remaining 66 documents underwent a comprehensive full-text eligibility assessment. In this phase, all 66 articles successfully fulfilled the strict inclusion criteria (such as geographic focus on Saudi Arabia and core AI technology integration), and consequently, no articles were excluded. After this systematic screening, these 66 core studies were officially included as the definitive basis for the qualitative systematic synthesis and the quantitative bibliometric mapping using VOSviewer software.

### 3.5 The bibliometric mapping and VOSviewer protocol

**Figure 1:**
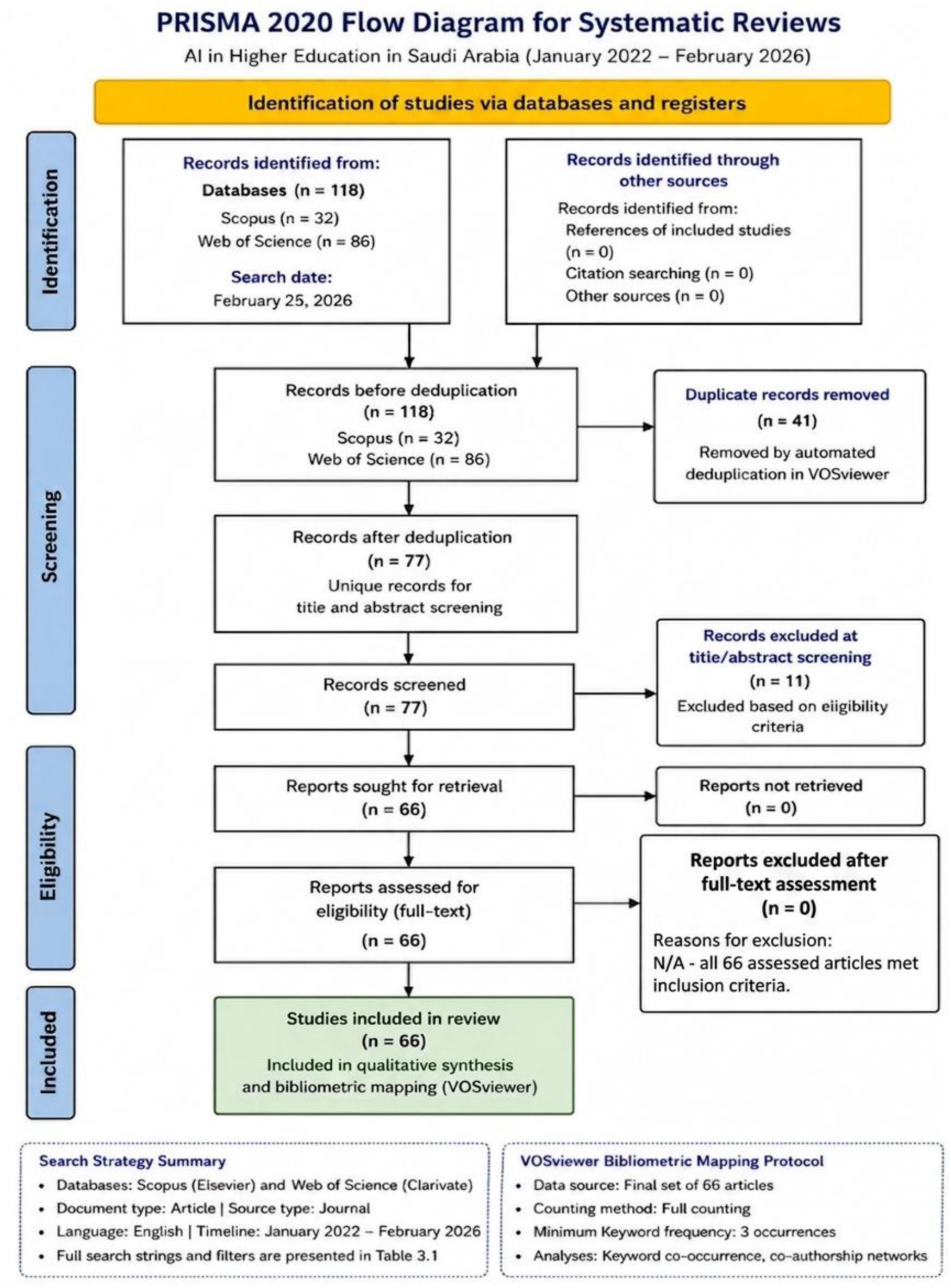
PRISMA 2020—systematic literature review (SLR) methodology.

The protocol for bibliometric mapping and VOSviewer, in order to supplsupplement the systematic review with a visual and network representation of the literature, a bibliometric mapping was also performed with the software VOSviewer. The last 66 articles were imported into VOSviewer for the analysis of co-occurrence in keywords and co-authorship network. A full counting method was used to make the bibliometric landscape fully reproducible, and the minimum number of occurrences for a keyword was set to three. The resulting network maps are an empirical illustration of the thematic clusters, prominent research fronts, and collaborative networks that characterize AI research in Saudi higher education, See Figure 1 PRISMA 2020.

**Figure 2:**
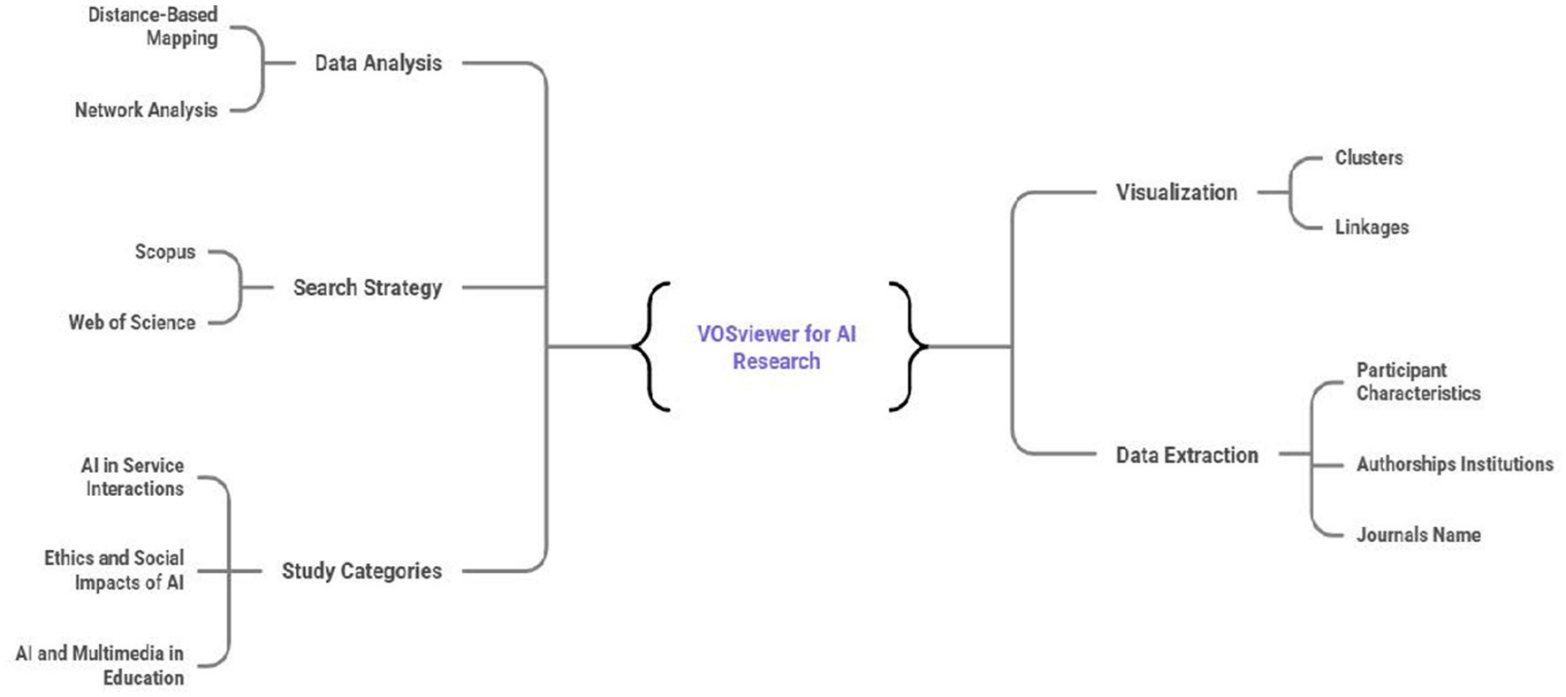
VOSviewer for AI in Saudi Arabia higher education research.

In addition, the VOSviewer software application was used to analyze and visualize the data in this research. VOSviewer is a powerful visualization software that uses a distance-based mapping method to visualize items (Ding and Yang, 2022). It displays findings in network and cluster formats in different colors, displaying the linkage, the strength of links, and the overall strength of links between the articles (Xie et al., 2020), the publication period of articles from January 2022 to February 2026 to analyze Systematic Literature Review – SLR. Full searches were performed in Scopus and Web of Science (WOS) (JCR/ESCI), as shown in Figure 2.

### 3.6 Results analysis and findings

**Figure 3:**
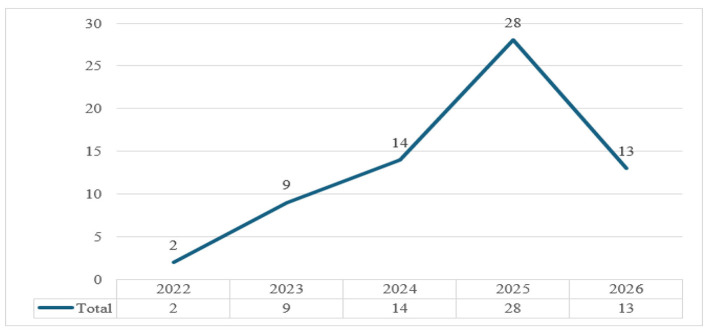
Descriptive analysis five-year publication papers timeline.

The descriptive analysis of the variables discussed in this section is the five-year publication period, the top five journals, authors, and Saudi Arabian-based research institutes. Figure 3 indicates that the number of publications is January 2022 to February 2026. Artificial intelligence in higher education Studies has been gradually growing since 2022 and will do so in 2024. The largest number of publications in recent year was in 2025, amounting to 28. The literature has depicted an increasing trend and wide concern for artificial intelligence within Saudi Arabia's higher education in both the education sector and academic setting.

Table 2 presents the top 10 research affiliations that published the highest number of papers on artificial intelligence in higher education. The top 10 research affiliations were King Abdulaziz University, Qassim University, King Saud University, King Faisal University, Prince Sattam Bin Abdulaziz University, Taibah University, Imam Abdulrahman Bin Faisal University, Imam Mohammad ibn Saud Islamic University, University of Ha'il, and Prince Sultan University. Two publications were recorded at King Fahd University of Petroleum and Minerals, King Khalid University, Majmaah University, Northern Border University, Arab Open University, Saudi Electronic University, and Princess Nourah bint Abdulrahman University. Meanwhile, Dar Al Uloom University, Effat University, Jouf University, Sulaiman Al Rajhi Colleges, Taif University, Umm al-Qura University, and the University of Bisha, with one publication each.

**Table 2:** Top contributing affiliations.

University name	No	University Name	No
King Abdulaziz University	9	Northern Border University	2
Qassim University	6	Arab Open University	2
King Saud University	6	Saudi Electronic University	2
King Faisal University	4	Princess Nourah bint Abdulrahman University	2
Prince Sattam Bin Abdulaziz University	4	Dar Al Uloom University	1
Taibah University	4	Effat University	1
Imam Abdulrahman Bin Faisal University	3	Jouf University	1
Imam Mohammad ibn Saud Islamic University	3	Sulaiman Al Rajhi Colleges	1
University of Ha'il	3	Taif University	1
Prince Sultan University	3	Umm al-Qura University	1
King Fahd University of Petroleum and Minerals	2	University of Bisha	1
King Khalid University	2	**Grand Total**	**66**
Majmaah University	2		

Source: Author (scopus and WOS database).

Table 3 lists the top 10 journals with the most published papers in the area of artificial intelligence in higher education in Saudi Arabia in recent years. More than 24 out of 66 articles recorded in the top 10 journals have been published in higher education sectors in Saudi Arabia in the field of artificial intelligence. Four papers have been published in the Sustainability Journal. In comparison, Acta Psychologica and Systems published six articles. The researchers then published 16 articles in Contemporary Educational Technology, Education Sciences, Frontiers in Education Future Internet, Humanities and Social Sciences Communications, International Journal of Interactive Mobile Technologies, Journal of Language Teaching and Research, and Theory and Practice in Language Studies. The rest of the study was written in a single journal per article, adding up to 40 articles. See Table 3. Moreover, each journal is ranked in Quartile Scores and indexed in Scopus and WOS.

**Table 3:** Top contributing journals.

Journals' name	No	Journals' name	No
Sustainability	4	Information	1
Acta Psychologica	3	Intelligent Systems with Applications	1
Systems	3	International Journal of Advanced Computer Science and Applications	1
Contemporary Educational Technology	2	International Journal of Computer-Assisted Language Learning and Teaching	1
Education Sciences	2	International Journal of Educational Sciences and Arts	1
Frontiers in Education	2	International Journal of Language and Literary Studies	1
Future Internet	2	Isagoge—Journal of Humanities and Social Sciences	1
Humanities and Social Sciences Communications	2	JMIR Medical Education	1
International Journal of Interactive Mobile Technologies	2	JOINETECH (International Journal of Economic and Technological Studies)	1
Journal of Language Teaching and Research	2	Journal of Decision System	1
Theory and Practice in Language Studies	2	Journal of Ecohumanism	1
AI and Society	1	Journal of Humanities and Social Sciences	1
Arabic Journal for Translation Studies	1	Journal of Information Policy	1
Australasian Journal of Educational Technology	1	Journal of Intelligent Systems	1
Cogent Business & Management	1	Journal of Intercultural Communication	1
Computers	1	Journal of Science and Technology Policy Management	1
Computers and Education Artificial Intelligence	1	Journal of the Faculty of Education, Tanta University	1
Cureus	1	Lecture notes in networks and systems	1
Discover Education	1	Pakistan Journal of Life and Social Sciences (PJLSS)	1
European Journal of Educational Research	1	Saudi Journal of Language Studies	1
Forum for Linguistic Studies	1	Scientific Journal of the Faculty of Arts – Assiut University	1
Frontiers in Artificial Intelligence	1	Scientific Reports	1
Frontiers in Psychology	1	Software Practice and Experience	1
Heliyon	1	Technological Forecasting and Social Change	1
IEEE Access	1	Tropical Journal of Pharmaceutical Research	1
Informatics	1	Grand Total	66

Source: Author (scopus and WOS database).

**Figure 4:**
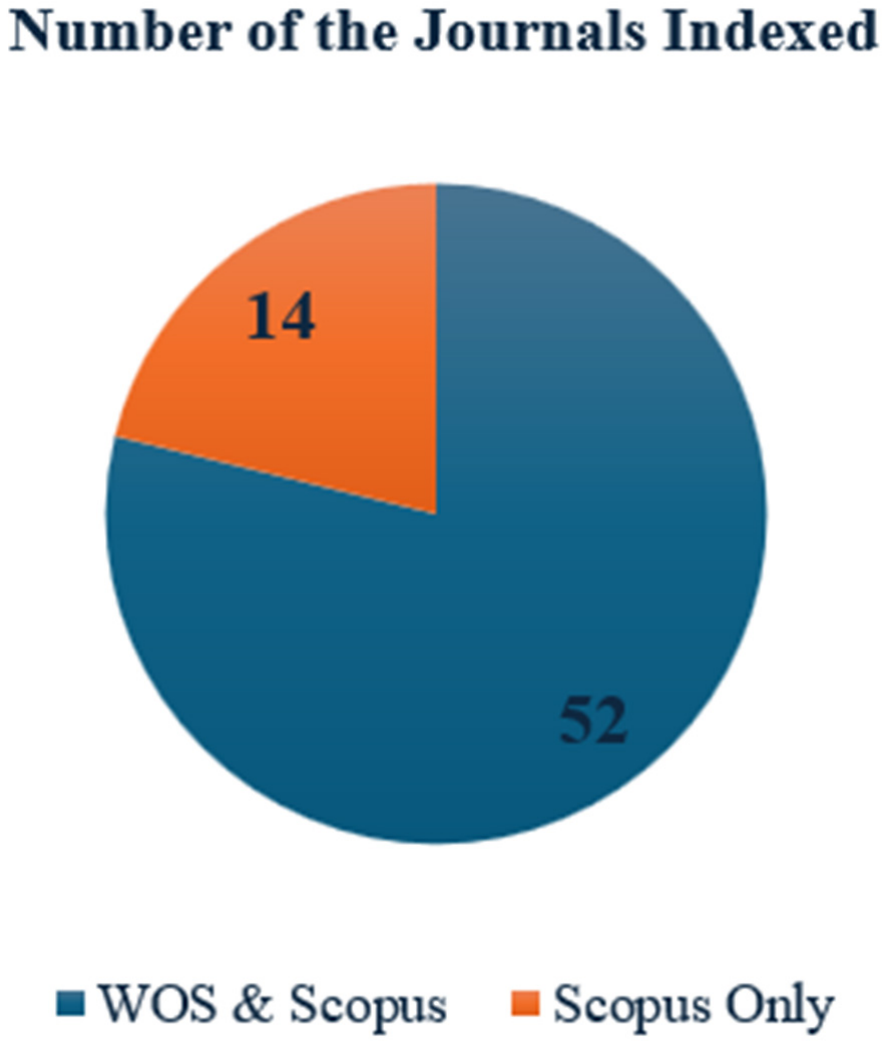
Number of journals indexed in Scopus and Web of Science WOS databases.

Figure 4 shows the count of journals indexed in Scopus and the Web of Science (WOS) database that published papers in the specific context of artificial intelligence in higher education in Saudi Arabia. The 52 papers have been published in WOS and Scopus indexed, and 14 papers have been published in Scopus indexed only.

**Figure 5:**
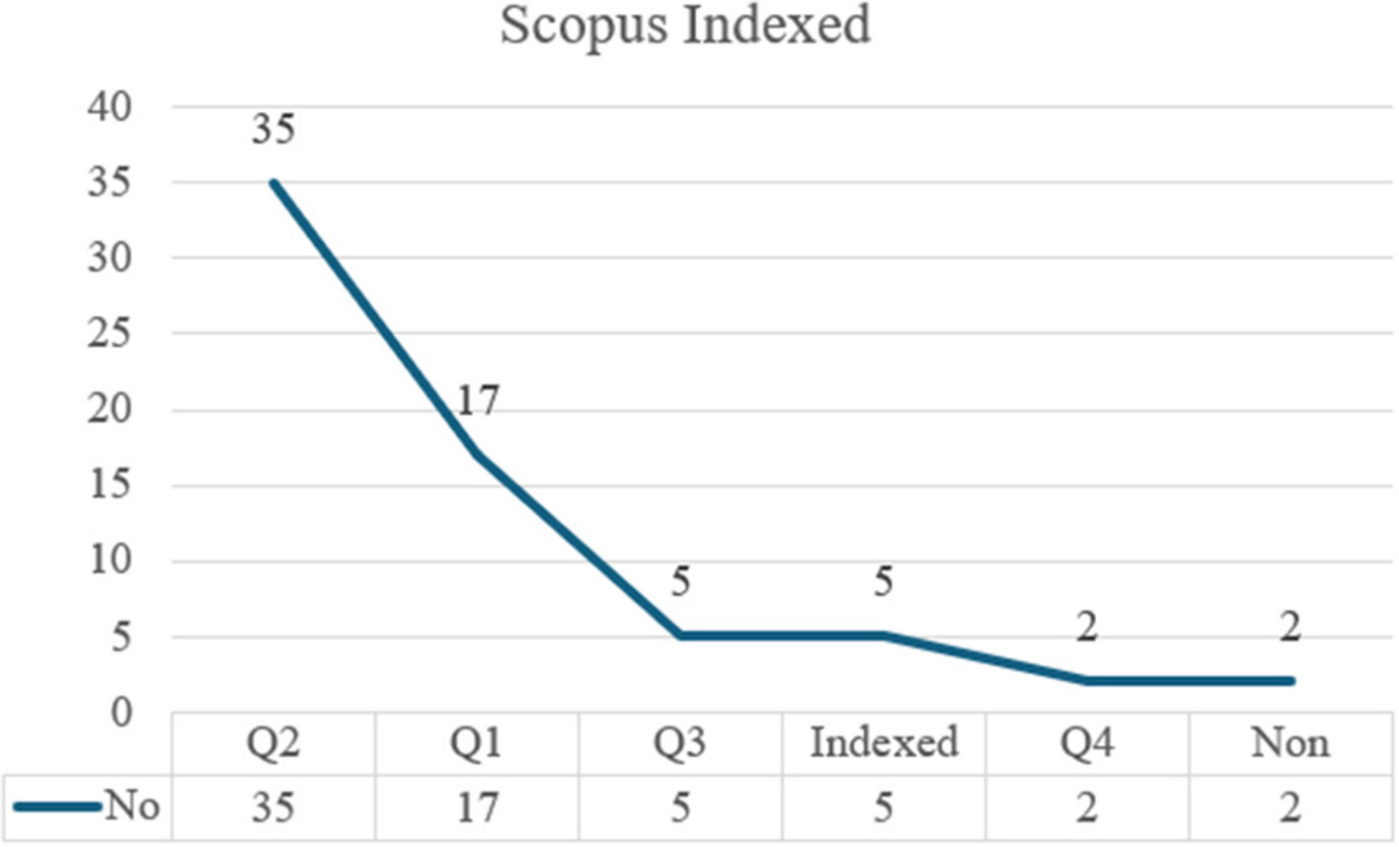
Top contributing quartile scores and were indexed in scopus. Source: author (scopus database).

Figure 5 shows the Quartile Scores and indexed the highest number of published papers in Scopus in the context of artificial intelligence in Saudi Arabia's higher education in the past few years. The 53 papers are published in Q2Q indexed, 17 papers are published in Q1Q indexed, five papers are published in Q3Q indexed, two papers are published in Q4Q indexed, five papers are published in indexed only without any Q, and two papers are published in non-indexed in Scopus.

**Figure 6:**
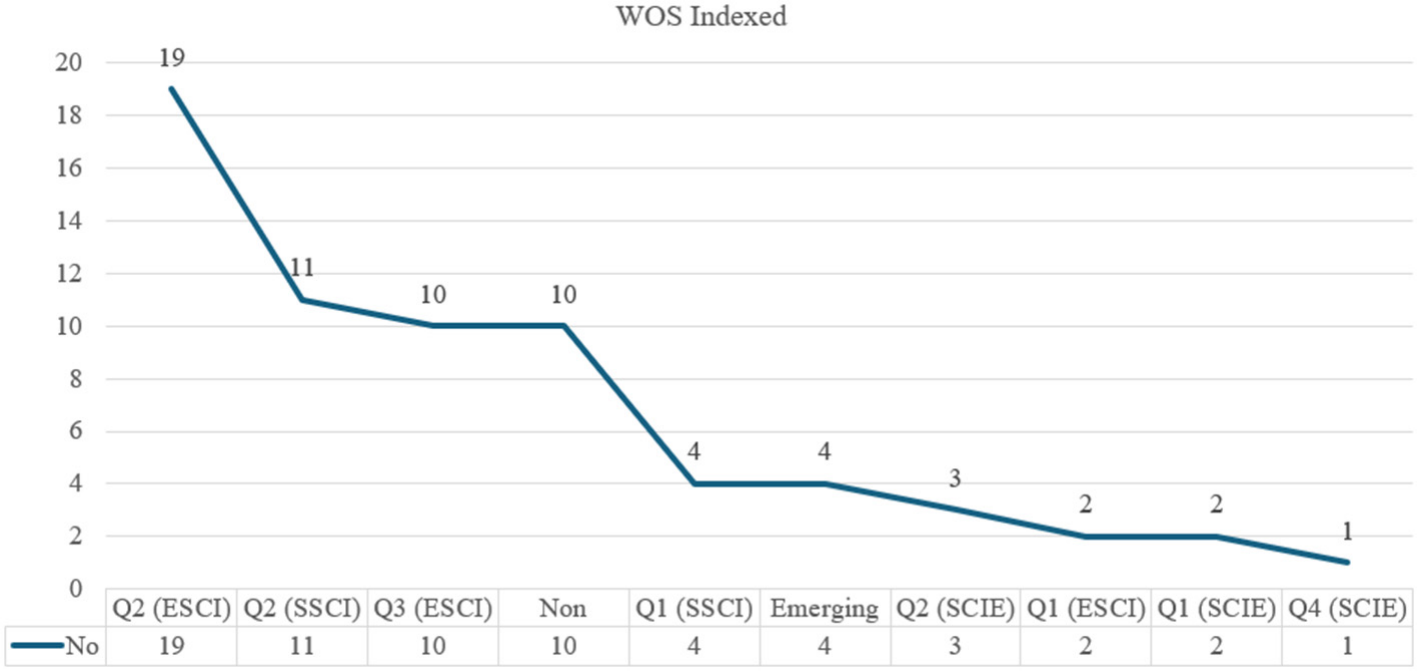
Top contributing quartile scores and were indexed in scopus. Source: author (wos database).

**Figure 7:**
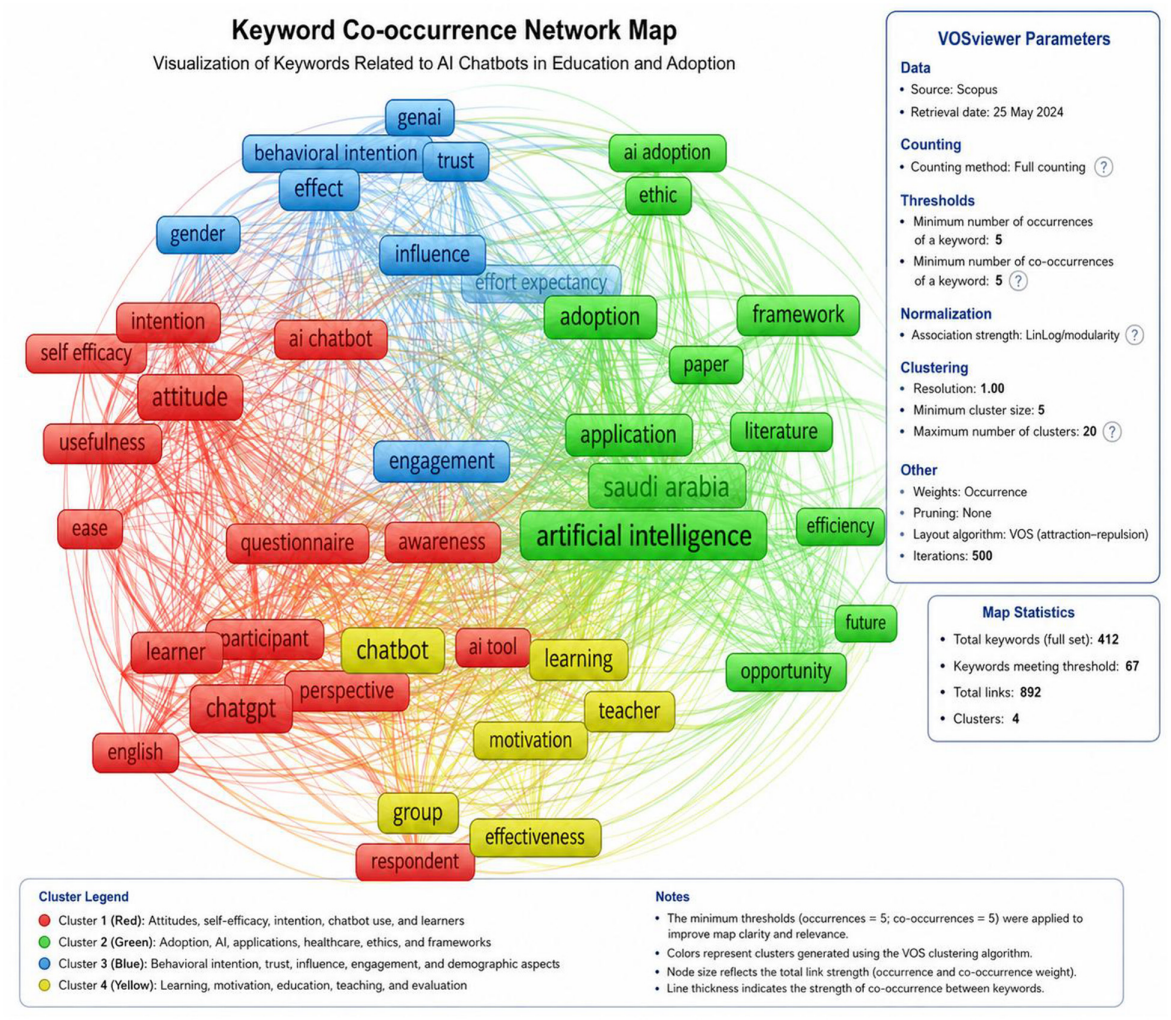
Network visualization of co-occurrence. Source: author (scopus, WOS database & VOS viewer software).

Figure 6 shows the Quartile WOS, indexed in WOS, with the most significant number of published papers only within the framework of artificial intelligence in Saudi Arabia's higher education in recent years. The 19 papers have published in Q2Q (ESCI) indexed, 11 papers have published in Q2Q (SSCI) indexed, 10 papers have published in Q3Q (ESCI) indexed, 10 papers have published non indexed WOS but it is Scopus indexed, four papers have published in Q1Q (SSCI) indexed, four papers have Figure 7 presents a network visualization of popular keywords based on co-word analysis. As expected, the artificial intelligence theme is a large cluster with a substantial connection to the higher education sector in Saudi Arabia, as indicated in Table 4. Moreover, five important personal clusters showed high correlations between different pairs of words. First, there is the high-education cluster that includes teachers, student learning, learning systems, Artificial Intelligence, AI Adoption, Framework, Application, AI Chatbot, and ChatGPT. Artificial intelligence supplements the ability of teachers to automate teachers, student learning tasks, and provide feedback on tailored education (Al-Rahmi, 2026a,b). Second, it supports student learning through flexible channels that meet diverse needs, increasing engagement, Learner, AI Tool, Teacher, Motivation, and Chatbot (Alharbi, 2025). AI improves learning platforms and information management through the analysis of large volumes of data, decision support, and improved institutional operations (Al-Mubireek, 2025). Third, AI allows data mining to determine tendencies in educational performance, and it helps colleges and universities to enhance their educational and administrative programs, GenAI (Generative AI), Behavioral Intention, Trust, English and Ethic (Al-Mamary and Alshammari, 2026). Fourth. The Saudi higher education cluster is linked to AI trends in data-driven decision-making, intelligent learning systems, and automation in education. Moreover, AI-based data mining enhances the process of curriculum design, and engineering education encompasses AI tools in simulations and automation, self-efficacy, usefulness, AI Chatbot, questionnaire, awareness, learning, and teacher (Almogren et al., 2024a,b).

**Table 4:** The bibliographic coupling analysis.

No	Year	Authorships institutions	Scopus	WoS (JCR/ESCI)	DOI links	Citation counts
1	2022	King Abdulaziz University	Q1	Q1 (SCIE)	https://doi.org/10.1016/j.techfore.2022.121772	125
2	2023	King Saud University	Q1	Q2 (SCIE)	https://doi.org/10.1109/access.2023.3314499	107
3	2023	King Faisal University	Q1	Q1 (SSCI)	https://doi.org/10.3389/fpsyg.2023.1129070	67
4	2024	Arab Open University	Q2	Q2 (SSCI)	https://doi.org/10.1080/23311975.2024.2348728	60
5	2023	Qassim University	Q2	ESCI	https://doi.org/10.36923/jicc.v23i2.125	57
6	2022	Princess Nourah bint Abdulrahman University	Q2	Q2 (ESCI)	https://doi.org/10.3390/informatics9040081	50
7	2023	University of Bisha	Q3	ESCI	https://doi.org/10.1108/sjls-10-2022-0072	43
8	2024	King Faisal University	Q1	Q2 (ESCI)	https://doi.org/10.30935/cedtech/15496	39
9	2024	King Faisal University	Q1	Q2 (SSCI/SCIE)	https://doi.org/10.3390/su162310780	30
10	2023	Taif University	Q1	Q1 (ESCI)	https://doi.org/10.1016/j.iswa.2023.200230	24
11	2023	King Fahd University of Petroleum and Minerals	Q2	Q2 (SCIE)	https://doi.org/10.1002/spe.3216	23
12	2023	Sulaiman Al Rajhi Colleges	Q3	Q3 (ESCI)	https://doi.org/10.7759/cureus.49486	23
13	2024	Prince Sultan University	Q2	Q3 (ESCI)	https://doi.org/10.1080/12460125.2024.2318527	19
14	2023	Effat University	Q1	Q2 (SCIE)	https://doi.org/10.1016/j.heliyon.2023.e20686	17
15	2024	Arab Open University	Q2	Q3 (ESCI)	https://doi.org/10.1515/jisys-2024-0237	16
16	2024	King Faisal University	Q2	Q2 (ESCI)	https://doi.org/10.3390/educsci14091034	16
17	2025	King Saud University	Q1	Q1 (SSCI)	https://doi.org/10.1057/s41599-025-04945-2	13
18	2024	King Abdulaziz University	Q1	Q2 (ESCI)	https://doi.org/10.3389/frai.2024.1398960	11
19	2025	King Abdulaziz University	Q2	Q2 (ESCI)	https://doi.org/10.3390/fi17020064	10
20	2025	King Faisal University	Q2	Q2 (SSCI)	https://doi.org/10.3390/systems13060490	10
21	2024	Qassim University	Q2	Non	https://doi.org/10.12973/eu-jer.13.2.573	9
22	2025	King Khalid University	Q2	Q3 (ESCI)	https://doi.org/10.3991/ijim.v19i14.56981	9
23	2025	King Abdulaziz University	Q1	Q1 (ESCI)	https://doi.org/10.1016/j.caeai.2025.100421	8
24	2025	Dar Al Uloom University	Q2	Q3 (ESCI)	https://doi.org/10.3991/ijim.v19i11.54643	7
25	2025	King Abdulaziz University	Q2	Q2 (ESCI)	https://doi.org/10.2196/76574	6
26	2025	Imam Mohammad ibn Saud Islamic University	Q1	Q1 (SCIE)	https://doi.org/10.1038/s41598-025-23447-4	5
27	2023	King Saud University	Q3	ESCI	https://doi.org/10.14569/ijacsa.2023.0140290	5
28	2024	Prince Sattam Bin Abdulaziz University	Q2	Q2 (ESCI)	https://doi.org/10.30564/fls.v6i6.7426	5
29	2024	Taibah University	Indexed	Peer Reviewed	https://doi.org/10.59992/ijesa.2024.v3n4p2	5
30	2025	Prince Sattam Bin Abdulaziz University	Q2	Q2 (ESCI)	https://doi.org/10.3389/feduc.2025.1669750	4
31	2025	Jouf University	Q2	Q2 (ESCI)	https://doi.org/10.3390/educsci15091136	4
32	2024	King Khalid University	Q4	Indexed	https://doi.org/10.57239/pjlss-2024-22.2.001449	4
33	2024	King Abdulaziz University	Q2	ESCI	https://doi.org/10.62754/joe.v3i3.3331	3
34	2025	Prince Sattam Bin Abdulaziz University	Q2	Q2 (SSCI)	https://doi.org/10.1016/j.actpsy.2025.105930	2
35	2025	Prince Sultan University	Q1	Q1 (SSCI)	https://doi.org/10.14742/ajet.10045	2
36	2025	Northern Border University	Q2	Non	https://doi.org/10.17507/tpls.1510.05	2
37	2026	Prince Sattam Bin Abdulaziz University	Q1	Q2 (SSCI/SCIE)	https://doi.org/10.3390/su18062674	2
38	2025	Majmaah University	Q2	Q2 (ESCI)	https://doi.org/10.4018/ijcallt.376344	2
39	2025	Qassim University	Indexed	Emerging	https://doi.org/10.59079/isagoge.v5i1.228	2
40	2026	King Abdulaziz University	Q2	Q2 (SSCI)	https://doi.org/10.1007/s00146-026-02858-5	1
41	2025	University of Ha'il	Q2	Q2 (ESCI)	https://doi.org/10.1016/j.actpsy.2025.106096	1
42	2026	Imam Mohammad ibn Saud Islamic University	Q2	Q2 (SSCI)	https://doi.org/10.1016/j.actpsy.2026.106244	1
43	2025	Imam Abdulrahman Bin Faisal University	Q2	Non	https://doi.org/10.17507/jltr.1605.19	1
44	2025	Imam Abdulrahman Bin Faisal University	Q2	Q2 (ESCI)	https://doi.org/10.3390/computers14110452	1
45	2025	King Abdulaziz University	Q2	Q2 (ESCI)	https://doi.org/10.3390/fi17100449	1
46	2026	Northern Border University	Q1	Q2 (SSCI/SCIE)	https://doi.org/10.3390/su18020785	1
47	2025	Majmaah University	Indexed	Emerging	https://doi.org/10.63939/ajts.91zp6t15	1
48	2025	Taibah University	Q4	Non	https://doi.org/10.1007/978-3-031-90998-6_2	0
49	2026	Prince Sultan University	Q2	Q2 (ESCI)	https://doi.org/10.1007/s44217-025-01089-y	0
50	2026	Imam Mohammad ibn Saud Islamic University	Q1	Q1 (SSCI)	https://doi.org/10.1057/s41599-026-06816-w	0
51	2025	University of Ha'il	Q2	Q2 (ESCI)	https://doi.org/10.1108/jstpm-06-2025-0252	0
52	2026	Qassim University	Q2	Non	https://doi.org/10.17507/jltr.1701.31	0
53	2025	Imam Abdulrahman Bin Faisal University	Q2	Non	https://doi.org/10.17507/tpls.1511.14	0
54	2024	Taibah University	Non / EKB	Non / EKB	https://doi.org/10.21608/aakj.2024.338876.1933	0
55	2024	Princess Nourah bint Abdulrahman University	Non / Arcif	Non / Arcif	https://doi.org/10.21608/mkmgt.2025.392173.1973	0
56	2025	Saudi Electronic University	Q1	Q2 (ESCI)	https://doi.org/10.30935/cedtech/17302	0
57	2025	King Faisal University	Q2	Q2 (ESCI)	https://doi.org/10.3389/feduc.2025.1711832	0
58	2026	King Abdulaziz University	Q2	Q2 (ESCI)	https://doi.org/10.3390/info17020157	0
59	2026	Qassim University	Q1	Q2 (SSCI/SCIE)	https://doi.org/10.3390/su18062986	0
60	2025	Saudi Electronic University	Q2	Q2 (SSCI)	https://doi.org/10.3390/systems14010031	0
61	2026	University of Ha'il	Q2	Q2 (SSCI)	https://doi.org/10.3390/systems14010065	0
62	2026	Umm al-Qura University	Q3	ESCI	https://doi.org/10.36892/ijlls.v8i1.2536	0
63	2025	Taibah University	Q3	Q4 (SCIE)	https://doi.org/10.4314/tjpr.v24i3.13	0
64	2026	King Saud University	Q2	Q2 (ESCI)	https://doi.org/10.5325/jinfopoli.16.2026.0004	0
65	2026	Qassim University	Indexed	Peer Reviewed	https://doi.org/10.59079/isagoge.v6i1.292	0
66	2025	King Fahd University of Petroleum and Minerals	Indexed	Emerging	https://doi.org/10.65479/joinetech.14	0

**Figure 8:**
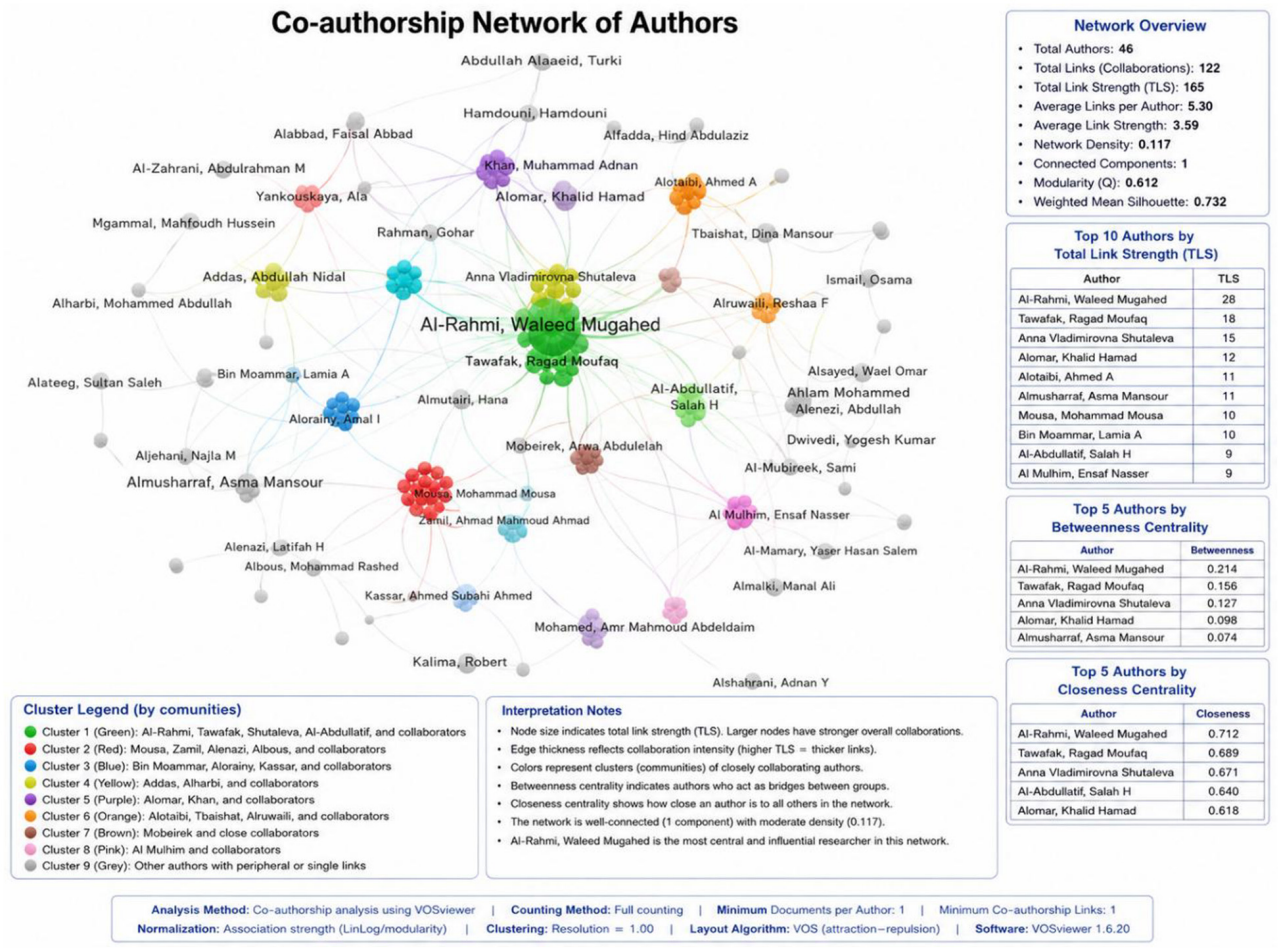
Network visualization of the most cited articles. Source: author (scopus, WOS database & VOS viewer software).

Figure 8 Citation Analysis depicts the network visualization of the top authors in terms of link strength of citation analysis and citation index. Citations are academic tools that help researchers evaluate the influence and importance of academic research in the field (Alwehebi, 2025). A high citation index usually indicates that a research publication has an impact on a field. In addition, the citation patterns of a publication may indicate the impact of research on scholars (Al-Rahmi et al., 2023; Sharmin et al., 2026). Meanwhile, more sophisticated tools, including genetic algorithms and deep learning, are not commonly used in web-based higher education. The PRISMA guidelines were adhered to in the study by Chu et al. (2022) to achieve transparency and reproducibility of the systematic review. The results of the study indicate that researchers mainly used AI technologies in the engineering field, especially in computer classes. AI has been used in profiling and prediction, intelligent tutoring systems, assessment, and evaluation.

**Figure 9:**
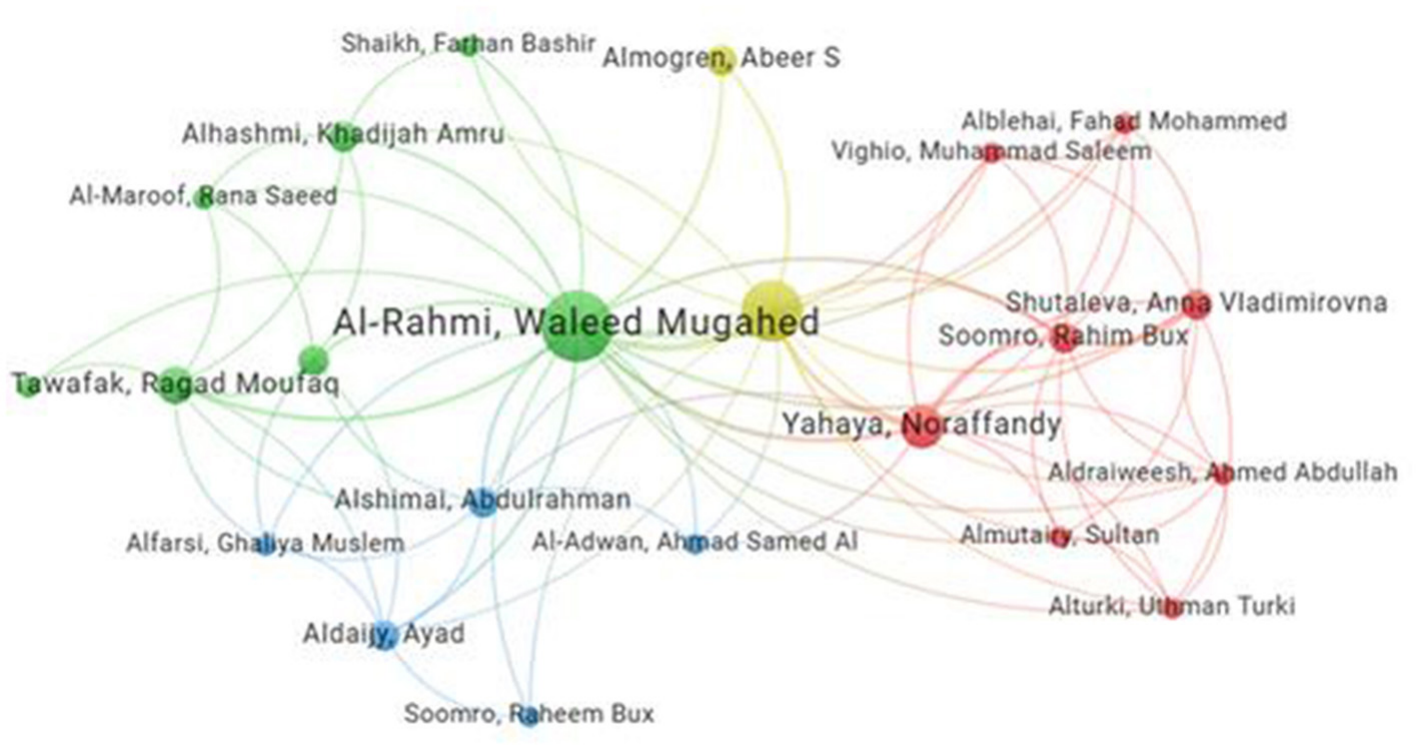
Network Visualization of authorship network global research collaboration: author (Scopus, WOS database & VOS viewer software).

Figure 9 shows the network mapping of the authorship network, illustrating global research collaboration based on the derivation of scientific publications. The network system highlights the existence of close connections between some nations, indicating that collaboration exists among academics. Saudi Arabia is a key country in international research networks and has created strong relationships with numerous nations, including the United Kingdom, the United States, Malaysia, India, China, Spain, Australia, and South Africa. Their influential position denotes a crucial degree of global co-authorship, likely because of their powerful research centers, funding opportunities and global influence (He et al., 2021). This means that Saudi Arabia influences academic networks, particularly in the social sciences and AI applied to education. Thus, within the context of AI in higher education, such collaborations are important for the development of AI-based educational improvements. Nations with robust research networks contribute to the creation of AI applications in learning management systems, adaptive learning, and intelligent tutoring systems (Al-Rahmi et al., 2026). Collaboration between technologically advanced countries and developing economies contributes to the transfer of knowledge and policymaking.

## 4 Discussion/implications

### 4.1 Bibliographic coupling analysis

As the bibliographic coupling analysis in Table 4 suggests, there is a clear and developing research collaboration pattern among Saudi universities and researchers in the fields of artificial intelligence, educational technology, and AI learning systems. The similarity in cited references among publications is the primary factor that affects coupling strength, suggesting that many publications are based on similar theories and recent publications related to AI. It showed that King Abdulaziz University, King Saud University, King Faisal University, Prince Sultan University, and Qassim University are the most productive, have better Scopus quartile rankings (Q1Q/Q2Q), and have relatively higher citation numbers, which means that these are the bibliographic coupling nodes. Institutions that showed better intellectual connections tended to reference similar foundational literature in their research on the adoption of AI and ChatGPT in education, the use of educational technologies, machine learning, and digital transformation. Further analysis reveals that a publication from a Q1Q or Q2Q journal is more bibliographically coupled than a publication from a lower-ranked or emerging journal. This implies that publication quality is likely to be similar in terms of theoretical frameworks, methodologies, and citation styles. Journals such as Technology in Society, Education and Information Technologies, Frontiers in Psychology, and Heliyon have a strong influence on the coupling structure owing to their high impact. Furthermore, the Coupling Network indicates a very narrow focus of research themes around the following:

- Artificial intelligence adoption,
- The adoption of ChatGPT and generative AI,
- Educational technology,
- Information communication technology (ICT),
- Motivation and AI's role in higher education.

The frequency of citations also suggests that older papers (20222–2023) receive more citations and have a greater scholarly impact than papers published more recently (20255–2026), likely because newer papers have not yet garnered enough citations. For instance, papers that garnered more than 50 citations are the intellectual hubs and are used as starting points for other more recent papers. Additionally, the bibliographic coupling structure indicates moderate to high thematic consistency among the items, indicating a growing convergence of the conceptual models adopted by researchers working in this field, such as:

- UTAUT Model,
- Unified Theory of Acceptance and Use of Technology (UTAUT),
- AI adoption frameworks,
- Theories of digital transformation and educational sustainability.

In summary, the bibliographic coupling analysis shows that the research field in Saudi Arabia is expanding and tightly connected, with significant institutional participation and increasing collaboration between researchers in the fields of artificial intelligence and educational technology.

### 4.2 Managerial recommendation

Overall, the above studies support the idea of the strategic integration of AI in Saudi EFL learning to increase its accessibility, effectiveness, and autonomy of learners (Alhalangy and AbdAlgane, 2023; Alharbi, 2025). AI applications such as ChatGPT have the potential to play a major role in language competence development in EFL classrooms in Saudi Arabia, but also require continuous refinement of the features of the tool to extract maximum benefits and usability in terms of education (Alwehebi, 2025). Thus, the given bibliometric analysis provides a more profound understanding of the development of artificial intelligence in the higher education literature as artificial intelligence studies develop. The increased importance of artificial intelligence within the higher education industry signifies the necessity of understanding the literature development and offering valuable feedback for future research. This study meets this goal by conducting descriptive research, which presents the necessary descriptive data and statistics in terms of the number of publications. The outcomes of the growth of publications and the top 10 journals, authors, affiliations, and universities may provide the necessary references to guide new researchers, university professors, and students in Saudi Arabia's higher education.

The second goal of this study was achieved through network analysis, which describes the expected connections between various components. The outcomes of co-word, citation, and bibliographic coupling processes point to the subjects, contributions, and future trends of research on artificial intelligence in the tertiary education industry. The outcomes of co-authorship research reveal collaboration patterns in universities. Several trends in research and collaboration have been identified as important for future research. Network analysis involves breaking down a particular set of words, including artificial intelligence, higher education, students, higher education institutions, artificial intelligence technology, ChatGPT, learning systems, and teaching. These phrases show the existing problems of artificial intelligence in Saudi Arabia's higher education sector.

- Future research can incorporate these factors in the formulation of hypotheses, correlations, or suggestions for further research. These trends are also opportunities for university lecturers to improve their research and teaching. These AI research trends can be utilized by students to create new projects, enhance the learning process, and seek new career opportunities in technology-oriented fields.
- Second, the findings suggest that researchers and affiliations in the artificial intelligence sector in higher education settings should enhance the research community by initiating partnerships between developed and emerging markets. The development of the research network and the strengthening of the field can contribute to the improvement of the existing knowledge of artificial intelligence in the sphere of higher education.
- Third, the development of AI research in tertiary education determines the authors who have had the most prominent impact on academic research. The lecturers were able to read the studies of the research author to enhance their available artificial intelligence guidelines, frameworks, and standards in the higher education sector.

### 4.3 Practical recommendation

The study proposes specific professional development for educators, culturally sensitive models in line with Saudi Vision 2030 objectives, and better design of AI tools to manage contextual and cultural peculiarities. The introduction of AI has the potential to develop critical thinking, learner motivation, and engagement, but must be implemented cautiously to prevent excessive dependency and misinterpretation of automated feedback (Alhalangy and AbdAlgane, 2023). The Saudi Arabia higher education Artificial Intelligence has various effects on various stakeholders, such as students, faculty, administrators, and policymakers, and has several challenges that should be carefully addressed. For students, AI can improve learning experiences by providing personalized learning systems, intelligent tutoring, and predictive analytics. AI-driven systems adapt content according to individuals' learning styles, enhancing engagement and academic outcomes (Alfaleh, 2026; Al-Maroof et al., 2025). However, issues of equity, information privacy, and surveillance are also major concerns. AI-based evaluation and surveillance technologies can generate ethical issues related to student autonomy and data confidentiality. The use of AI should be transparent and have clear data protection policies that guarantee the trust and wellbeing of students.

Nevertheless, faculty members usually struggle to adjust to AI technologies, primarily because they are not technically trained and fear losing their jobs to AI. Other teachers are also worried that automation will crowd out human teachers in the learning process of students (Al Faia and Alomar, 2025). To solve this, institutions ought to focus on AI literacy courses and professional development programs that will enable faculty to use AI in their teaching.

Predictive analytics can be used to predict students who are likely to drop out, and the university can implement early intervention measures to ensure that such students are supported. It is also through AI that operations through the automation of admissions, financial planning, and scheduling, which enhances efficiency (Alqahtani and Alrwais, 2023). Nevertheless, the use of AI in decision-making provokes issues of bias in algorithms and the possibility of unbiased administrative procedures (Al-Rahmi et al., 2026; Soomro et al., 2025). To make institutional decisions based on AI fair and accountable, mechanisms of oversight that focus on ethical governance and transparency are necessary. Policymakers can contribute to the integration of AI into higher education by developing regulations that encourage ethical adoption without deterring innovation. AI may assist policymakers in making evidence-based policies, including processing mass data on student performance, determining points of curriculum change, and anticipating future trends in education (Alhalangy and AbdAlgane, 2023). Nevertheless, it is not easy to provide equal access to AI technologies among people representing various socioeconomic groups.

The high costs of AI implementation and the absence of digital infrastructure are problems in many universities, especially in developing countries. To eliminate these differences, it is necessary to invest in AI education at the public level, provide funds to adopt AI in institutions, and create standard regulatory frameworks. To implement responsible AI usage in higher education, universities must establish governance mechanisms to safeguard against bias, fairness, and accountability in AI-based decision-making. Professional development programs should include faculty training and AI literacy activities to ensure that educators have the required skills. Institutions should also implement data protection policies that are in line with international privacy laws to protect students' information. Policymakers should aim to create uniform policies that guarantee the ethical use of AI without undermining interdisciplinary research and practice between academia and industry. When developing scalable solutions to support students in low-resource universities, researchers should examine the long-term effects of AI on student learning, faculty work, and institutional decision-making.

The papers propose conducting more studies in different regions and fields to further legitimize the research and encourage the use of the digital infrastructure of Saudi Arabia to improve AI-assisted language learning (Al-Rahmi al., 2023; Alwazzan, 2024; Alshammari and Alshammari, 2026; Qasem et al., 2023). Furthermore, this research highlights the need to encourage positive attitudes and familiarity with AI tools to ensure that they are utilized to their fullest potential in the Saudi setting (Alsaedi, 2025; Alfarsi et al., 2024). The results indicate that the barriers to the potential of chatbots in education should be overcome by educators and policymakers to benefit from the full potential of chatbots in education (Alsayed et al., 2024). This will help uphold academic integrity and enhance the quality of research with the growing use of AI (Alshaibani et al., 2025; Al-Rahmi et al., 2026). This is in line with the objectives, outcomes, and findings of this study.

## 5 Conclusion

This study provides a comprehensive analysis of AI in Saudi Arabia's higher education through bibliometric and content analyses, which present important information about its evolution, applications, and future. The present bibliometric analysis utilized the Scopus database, which is a scientific database that can be considered one of the most comprehensive sources of peer-reviewed literature. As part of the title article search, the search terms Artificial Intelligence, AI tool, and Higher Education were added on January 1, 2026, leading to the inclusion of 128 documents published between January 2022 and February 2026. After identifying and eliminating duplicates, as well as editorials, notes, and reports, we narrowed the dataset to 66 articles. Two main forms of bibliometric analysis were applied to the current research, including descriptive (publication trend, contributing universities, affiliations, authors, and journals) and network analysis (co-word, citation, co-authorship, and bibliographic coupling analysis) and content analysis of the most cited articles on AI in higher education. In this study, the VOSviewer software application was used to analyze and visualize the data.

The findings show that research outputs have increased significantly, particularly after 2022, proving the increasing interest in the role of artificial intelligence (AI) in education in Saudi Arabia. This growth has been driven by advances in artificial intelligence technology, the digital revolution accelerated by the COVID-19 pandemic, and greater global investment and collaboration. King Abdulaziz University, Qassim University, and King Saud University have made their mark as front runners in the use of AI. Respected journals, such as Lecture Notes in Networks and Systems and Education and Information Technologies, have played a significant role in sharing the necessary research. Adaptive learning systems, intelligent tutoring, predictive analytics, and administrative optimization are thematic areas of artificial intelligence (AI) in higher education. Key topics and keywords, such as artificial intelligence, higher education, students, and ChatGPT, underline the incorporation of artificial intelligence (AI) into the system to provide specialized education, proper management of resources, and implementation of innovative technologies. Co-word analysis presents interconnected clusters that explore ethical concerns, engagement education, and e-learning developments in Saudi Arabia.

References to notable articles demonstrate both the beneficial role of artificial intelligence (AI) in enhancing the educational process and the ethical problems, including the presence of biases, data security, and the loss of human-centered approaches. Despite its transformational potential, artificial intelligence (AI) in Saudi Higher education faces serious challenges. Ethical issues, limited access to advanced technology, educator preparedness, and institutional adaptability remain serious issues. These challenges involve multidisciplinary collaboration, partnerships in the international arena, and exploration of more sophisticated AI applications, including deep learning and generative artificial intelligence (AI). Therefore, some practical recommendations of this study are as follows:

- Institutions should have clear ethical standards that would help maintain fairness, transparency, and accountability in AI applications, and invest in AI literacy programs so that educators and students can gain the necessary skills.
- Interdisciplinary research partnerships between AI professionals, educators, and policymakers should be encouraged as a solution to overcome adoption issues and enable innovation.
- To enhance inclusiveness, universities should ensure that there is no infrastructure gap, especially in the developing world, so that all people have fair access to AI technologies.

More studies should be conducted to understand the effect of AI on student engagement, student learning, and administrative processes to inform its successful adoption in higher education in Saudi Arabia.

Some of the limitations of this study include the use of Scopus and Web of Science database that could have omitted other studies of interest that are registered in other databases like Google Scholar, Crossref, Doaj and pubmed. Furthermore, excluding non-English language publications on Arabic narrows the perspectives of AI research globally within Saudi Arabia. Although bibliometric and content analyses can be useful, they cannot reflect the practical issues of AI implementation in higher education, including institutional resistance and student experience. Qualitative approaches, such as case studies, interviews, and surveys, should be used more effectively in future research to learn about the real-life implementation of AI in various learning settings. Saudi Arabia should also be compared with other Arabian Gulf or Middle Eastern countries.

Regarding the screening process, all title, abstract, and full-text assessments were conducted by the author using a predefined and clearly specified eligibility framework established prior to the search process. This approach ensured consistent and uniform application of the inclusion and exclusion criteria across all review stages. Nevertheless, as with many evidence synthesis studies, the use of a single reviewer may be considered a limitation of the review process. Future research can build upon this work by incorporating multiple reviewers to further enhance the breadth of perspectives and strengthen procedural robustness.

## Data Availability

The original contributions presented in the study are included in the article/supplementary material, further inquiries can be directed to the corresponding author.
